# Supplementary material for: Chromothripsis during telomere crisis is independent of NHEJ, and consistent with a replicative origin
Source: Genome Res. 2019 May;29(5):737–49. doi: 10.1101/gr.240705.118 (PMC6499312; doi:10.1101/gr.240705.118)
Supplement: Supplemental Material [file supp_gr.240705.118_Supplemental_file_1.zip › contigs/annotated_contigs/DB75/contig.3.DB75_length_556_mean_cov_22.7517985612.docx]

**DB75_length_556_mean_cov_22.7517985612**

```
TTACAAAAAAAGCACTTGTCATGT|TTTTGAATTTCTATATGCAAAAAGTTACGACATATTTAAAGTCTAAACAGAGACATAGGATGGC
                         >chr13:63632871-63633116 - E=9e-77
TTAAGAAAACCAAAACGCTTACCTGAATAGATAAAAATAAAGGCCATAAATCATAATAACAATAAAAAACACAAATGCAGAGAAGTAAG

ATAGTACTGTAACTGGTATTAACACTGAGAACATCTGTTGAATACTTTTGGCCTAGAACTGAATCTTAGTGGGTATATATATAT|TTTT

TTCCT|TTTCTTTTTTTTTTTTGCACAAATTGAGAGATAAACTACCAAACCTGGAACCTACAAAGGGTGAAAGGTTAGTTGTTTAGAGA
      >chr13:63632702-63632824 - E=3e-38 p=3e-02
CGAGACCCCACAGGGAATGATCTCCTGAGTGCACAAAAATGC|CCACTTGTTAACAAAAACG|AAAGATAATGTATTTGCCCATATTGA
                                                              >chr13:63632547-63632689
CAATAATATTGAGTAGAAATCAAAAATAAAAGCTAAAAGTCTCCACTAAGTTTCTAGAGACACAATCTAATTGTTACCGGTGGAGCGTG
- E=1e-61
TCCGGATCCTTGGCGTTTTGAACAAAG
```
